# Porcelain as an Art in Crown Work

**Published:** 1901-08-15

**Authors:** F. J. Capon

**Affiliations:** Toronto, Can.


DENTAL REGISTER.

Vol. LV. August 15, 1901. No. 8.

### PORCELAIN AS AN ART IN CROWN WORK.

BY F. J. CAPON, M.D.S., D.D.S., TORONTO, CAN.

Abstract of paper read before the Tri-State Dental Meeting, Indianapolis, Ind., June, 1901.

Porcelain has come to our aid as an ideal material for the replacement of the lost crowns of teeth in conspicuous places; it has the required strength, and when applied with a true sense of art gives results that please the operator, the patient, and deceives the public.

Too many crowns are made that do not fill the interdental space, and are a source of great annoyance to the wearer.

For the six anterior teeth, particularly if the patient be a lady, it is desirable that the festoon of the gum be kept in a normal condition, that no one should suspect the artificiality. A semi-band is indicated here rather than the entire band, which is apt to interfere with the nice results at the labio-gingival margin, causing irritation, resulting in recession. We are forced to admit that bands of the neatest character are more or less liable to induce this condition. For many years partial banding has given me very great satisfaction.

ROOT PREPARATION.

The root preparation for a crown of any style is important; many failures may be attributed to improper preparation. The face of the root should be ground to a wedge shape following the gum line without wounding the soft tissue; remove the remaining ledge of enamel, and with an Evans trimmer cut the edges of the root just under the free margin of the gum, making the face of the root more of a cone than a wedge; enlarge the canal to receive a tapering, square iridio-platinum wire, the depth of the canal is marked on it, then it is pushed through a disk of platinum a little larger than the face of the root, and soldered at the mark with pure gold or platinum solder. With a flat-faced burnisher work the platinum disk down to the face of the root, carrying the burnisher well along the edges, which will be defined on the platinum; remove and trim away the surplus, leaving sufficient on palatine half to form the band, which is turned approximately to line of root with small, nosed pliers; replace it on the root and burnish it again until the platinum lies snugly to the face of the root and the banded portion closely in position.

MAKING THE CROWN.

The facing is ground to nicely fit the labial surface and stand in its proper relation, the pins are bent to touch the head of the post and facing kept in position with hard wax; it is then removed from cast, invested, and soldered with the minimum amount of pure gold, or better, platinum solder. If a surplus is used in soldering, it will cause porosity in the porcelain, which is detrimental to the strength, appearance and finish of the crown; this necessitates that in all porcelain work it is essential that all the metal parts to be united should have absolute contact, and any surplus end of dowel or pins should be ground and made round to void sharp corners. High grade bodies should also be used in crown work, the higher the fusing point of the body the stronger the crown. The glass bodies used by those seeking low fusing point have done much to depreciate artistic work, as these bodies have caused a succession of failures in both crown and inlay work.

The application of the porcelain body should be done with clean surroundings; a fresh towel or white paper spread upon the bench or table, and any work going on in the laboratory that would create a dust should be stopped; these precautions will insure against minute particles of carbon settling and leaving tiny black specks on the finished porcelain surface. By holding the crown in tweezers or pin vise, the porcelain is thoroughly worked down into every crevice; by rapping the tweezers the moisture is brought to the surface and taken up by old linen, bibulous or blotting paper.

The crown is now given its first baking and tried in the mouth and trimmed or built up to perfect contour and occlusion, and then given the final or hard baking. Two bakings are sufficient for this class of work, but sometimes a flaw or mistake will necessitate a third baking. The crown is finally finished by sandpapering the edges and any necessary polishing.

The crown just described can have a full band or cap if the case demands it. No better crown could be suggested for a man, but for a lady the half band is ample for all the requirements with a more esthetic appearance; and after many years, I have no reason to regret their use where properly indicated.

### CROWNING BROKEN-DOWN TEETH.

In those roots that are badly decayed below the margin, the root of one may be used for a crown for esthetic effect alone. The root is brought to a condition of assured health, and the apex of the canal sealed. It is now required to restore the root form by means of a rigid and insoluble material; amalgam is best adapted for this purpose. If the edges are easily accessible, the root can be dried, the canal cleaned and tapped for the reception of a gold screw-post; after placing a small piece of cement on its extremity it is screwed into place. The root is given an undercut if possible to aid in retaining the amalgam, which is packed about the pin, over the face of the root to form a slight cone. If the root is fractured or decayed considerably below, then a matrix with an apron to take in the extent of fracture is required in which the amalgam is to be packed. The flow of blood is invariably bothersome at this point, but can be nicely controlled by using *adrenalin chlorid* as a stiptic and cocain as an anesthetic. The size of the band or tube matrix can be obtained by packing a small cylinder of moldine over the root end; while the moldine is in position a small impression tray filled with the same material is used to secure and withdraw the mass covering the root face. Into this impression a fusible metal cast is run, on which the matrix of German silver is adjusted, fitting the root accurately. If the root is that of a bicuspid or a molar, one on which a collar crown is to be placed, the matrix is made deep enough to grasp the root firmly and to project about an eighth of an inch above the margin of the gum. If a tube crown is to be used it is finished to the gum margin, leaving the screw posting projecting. After twenty-four hours the matrix can be removed by splitting it with a hatchet excavator ; Rhein files are then passed down between the gum to remove any rough edges of amalgam. The matrix in some cases is made of thin platinum and allowed to remain, covering the amalgam mass. In either case the external edge of amalgam can be ground down to suit the kind of crown desired.

To obtain the tube for the crown, wrap a piece of thin platinum around a piece of similar posting, remove the tube and place it on the dowel head ; then take a disk of soft platinum, No. 35 gage, somewhat larger than the face of the root ; make a hole in the center of it and force it over the tube which is in position on the dowel head ; with a pair of tweezers pinch the torn flanging edges (of the hole) against the tube, remove the tube and disk together and solder with minimum amount of platinum solder. Now there will be a tube and disk instead of the dowel and disk.

The surplus tubing is bitten off at the length of the screw post, and the procedure of the balance being the same as the former crown.

### THE CUP CROWN.

The "cup" crown for bicuspids and molars is made with or without a dowel, as the case demands. The root is prepared as for any banded crown, but when no dowel is used a little more tooth tissue should be left for retention on the palatine aspect.

A platinum band (which for porcelain should always be beveled and lapped at the joint) with plenty of width is made to fit the root accurately and festooned so that it will not lacerate. While in position on the root, scratch with a pointed instrument on the inside of the platinum band the line of the root, also the gum line on the outside of band. With a pair of fine-pointed scissors cut the buccal face of band to line of the gum, and back far enough so that no metal will show when the crown is completed; cut and fit a floor to the band of No. 30 gage platinum, which is soldered to place. Trim off any surplus and file the buccal edge, rounding which allows of a better extension of porcelain on the band. If a dowel is to be used it can be adjusted and soldered in its proper relation at this stage. The extended portion of the band is contoured if necessary and the crown proceeded along the same line as the former ones; if a dowel is used the pins of the facing are soldered to it, if no dowel the pins are bent down and soldered to the cap or floor of cup.

The band projecting above the face of the root forms a "cup" in which the porcelain has its base and gives the additional strength required for mastication, the liability of fracture of porcelain being reduced to a minimum. The facings of these crowns give little cause for anxiety as they are made of "block" body, the entire surface of which is etched to the body of the crown; it is soldered as well. This style of porcelain crown can be used to serve as abutments of gold bridges as they have a soldering surface.

### DISCUSSION.

Dr. J. E. Nyman, of Chicago: The essayist makes a strong appeal to the artistic side of our practice as a profession. He is endeavoring to restore natural conditions, and to do it in such a way that none can detect the art. It is this that makes us a profession. It is only as artists that we give anything to the world. The idea of building a crown without mutilating the gum, and of such form that it harmonizes with its environment and preserves the interproximal space is the work of an artist and not the mechanic only. Illy-fitted crowns cause diseased conditions of the gums and are pernicious. Esthetic crowns give pleasure to the patient as well as comfort. The preparation of the root end is important, as the essayist says. Many dentists destroy the soft tissue by unnecessary mutilation with instruments; care should be exercised in using disks to grind the periphery of the stump as the abrasive material works into the soft tissues and can not be removed except by suppuration and loss of valuable structure. I have used with great satisfaction Dr. A. G. Johnson's enamel cleavers. I trim the stump end below the gum margin by burring out with a round bur the root, being careful not to touch the gum, then take a sharp chisel and cut down the thin peripheral margin to the desired point

I do not believe it is good practice to trim a stump down close to a live pulp; it is much better to devitalize and remove the pulp, and then trim the stump to the best advantage.

The character of the work will determine whether to use a full or partial band. In some cases the partial band will suffice. I sometimes make partial bands for bicuspids, more often for incisors.

The post should not be too long. In most cases it serves no good purpose. It should be only long enough to be firmly secured in the tooth substance.

In selecting colors for crown it is not always best to match adjacent teeth, but corresponding teeth in the same jaw, and avoid getting shades too light.

For investment material I have had excellent success with Brophy's investment compound, it stands the fire better than any I have used. Don't pour boiling water on any investment to clean out wax as it will ruin the investment.

I have recently been using Brewster's body with great satisfaction; it is strong and high fusing and can be nicely polished. Distilled water should always be used in mixing body to prevent discoloring.

Dr. H. J. Goslee, of Chicago: The essayist has given us a good paper. I would suggest a slightly different stump preparation than that which he gives and which is generally used. I would allow the lingual side of the stump to stand as much as is practicable above the gum margin, and cut the labial side sufficiently beneath the gum margin to hide the joint and all metal. Then I would cut off the stump on a straight line from the labial to lingual preparation giving to the end a straight bevel instead of an angular one as usual, with the angle at about the position of the post. My preparation gives good support to the crown and makes it much easier to manipulate the materials used. It is a good idea not to dress the stump end as much as necessary until the peripheral preparation has been made, so as to avoid lacerating the gum unduly.

Porcelain crowns are much stronger than soldered metal and porcelain crowns, for the reason that no metal crown is stronger than its weakest part which is the two small pins baked into the porcelain. Porcelain crowns are more artistic, as they can be made to harmonize with their surroundings. Some facings will change color in the high heat necessary to use porcelain, but there are makes which will not change. I usually use a full band for stability, although a partial band may be used where a special emergency seemed to demand it, and where the strain upon it would not be excessive.

I don't agree with the essayist's method of burnishing the cap material down upon the sides of the stump to form a band. I much prefer cutting out and soldering a band of heavier material than the cap.

Should I encounter difficulty in retracting the gum from the root end, I make a temporary crown with a vulcanite tooth and a German silver post soldered with soft solder and insert it with gutta-percha and have the patient wear it a few days and it will then give perfect freedom for manipulation.

In making bicuspid crowns, instead of making the cup shape band of the essayist, I solder a strong post on the cap behind the facing where the lingual cusp is to be placed; this will prevent this cusp from being broken off in mastication.

I prefer to use round or oblong posts as the porcelain material can be packed around them easier.

